# Alternative Splicing Increases Sirtuin Gene Family Diversity and Modulates Their Subcellular Localization and Function

**DOI:** 10.3390/ijms22020473

**Published:** 2021-01-06

**Authors:** Xiaomin Zhang, Fathima S. Ameer, Gohar Azhar, Jeanne Y. Wei

**Affiliations:** Donald W. Reynolds Department of Geriatrics and Institute on Aging, University of Arkansas for Medical Sciences, Little Rock, AR 72205, USA; FSAmeer@uams.edu (F.S.A.); AzharGohar@uams.edu (G.A.)

**Keywords:** novel isoforms, exon skipping and inclusion, age-related expression, mitochondrial function

## Abstract

Alternative splicing generates multiple distinct isoforms that increase transcriptome and proteome diversity. There are seven sirtuin genes in humans, each consists of multiple exons that are likely to undergo alternative splicing. Our aim was to characterize the effect of alternative splicing on the sirtuin genes. Here, we report the identification of 23 human sirtuin isoforms, most of which were not previously reported. Five of the sirtuin genes had more than one isoform, whereas sirtuin-6 had nine isoforms. Exon skipping was the main event. Most of the sirtuin isoforms were deficient in parts of the protein domains, including the catalytic domain, the N- or C-terminus, nuclear localization signal or mitochondrial targeting signal. The domain loss caused potential structural changes. Three SIRT1 isoforms had a differential effect on the mitochondrial oxygen consumption rate. Age-related changes in the expression of SIRT1 isoforms were observed in the human heart in fetus, adults, and very old individuals. We also identified 15 sirtuin isoforms in mice. Our data indicate that alternative splicing increases sirtuin gene diversity and may modulate subcellular localization and function, thereby adding complexity to the gene regulation of mitochondrial respiration, metabolism, and cardiac function during maturation and aging.

## 1. Introduction

The sirtuin proteins belong to the class III histone deacetylases (HDACs), which are NAD^+^-dependent enzymes that deacetylate numerous proteins, including histones and other non-histone proteins. The sirtuin-1 (SIRT1) gene is the founding member of the mammalian sirtuin family. Its homology with yeast Sir2 was initially discovered in the position-effect control of yeast mating. Subsequently, it was found that the yeast Sir2 gene plays an important role in cell cycle progression, gene silencing, and chromosome stability [1]. Seven mammalian homologs have been identified (SIRT1 to SIRT7) in mammals [2]. They share a conserved central catalytic domain, which is approximately 250–270 amino acids in length among the seven genes. The N- and C-terminal sequences are diverse, which host the sequences for distinct subcellular localizations, including nuclear localization signal (NLS) and mitochondrial targeting sequence (MTS). Each sirtuin protein enters a specific intracellular compartment where it deacetylates its target proteins. Among the seven sirtuins, SIRT1, SIRT6, and SIRT7 are usually localized in the nucleus, where they regulate gene expression by changing the acetylation status of histone proteins and chromatin structure. SIRT2 is localized mainly in the cytoplasm, where it deacetylates cytoplasmic proteins, including α-tubulin that maintains cellular architecture and morphology. SIRT3, SIRT4, and SIRT5 are often located in the mitochondria where they interact with non-histone proteins and deacetylate proteins that are involved in fatty acid oxidation, tricarboxylic acid cycle, and oxidative phosphorylation [3,4,5,6]. Therefore, the sirtuin family proteins exert their diverse functions at various cellular locations and regulate processes, including metabolism, stress response, growth, and aging. 

Alternative splicing occurs in transcripts produced by more than 95% of human genes [7,8], including ones implicated in metabolism, senescence, apoptosis, and DNA repair [9,10,11]. During the splicing, pre-messenger RNA is processed to remove introns and to include or exclude certain exons, thereby generating multiple distinct isoforms from a single gene [12]. Each sirtuin gene contains multiple exons and is likely to undergo alternative splicing, which will generate isoform variant(s) that may have a different biological function. 

In this study, in an effort to define the effect of alternative splicing on sirtuin gene-product diversity, an approach combining bioinformatics analysis and screening of RT-PCR fragments and clones was employed. The results showed that there are twenty-three sirtuin isoforms in the human genome and fifteen sirtuin isoforms in the mouse genome. Experimental data demonstrated that both exon skipping and exon inclusion occurred in sirtuin-1 (SIRT1) gene. The SIRT1 (SIRT1-v1) protein was localized in the nucleus, whereas two new SIRT1 isoforms, SIRT1-v2 and SIRT1-v3 lacked NLS signal and localized in the cytoplasm. The three SIRT1 isoforms had differential effects on the mitochondrial oxygen consumption rate. An age-related change in the SIRT1 isoform expression was observed in the hearts of human fetal, young adult, mature adult, and very old individuals. These data indicate that alternative splicing increases the sirtuin gene family diversity, which adds complexity to the gene regulation of mitochondrial respiration, metabolism, and cardiac function during maturation and aging.

## 2. Results

### 2.1. Twenty-Three Sirtuin Isoforms in the Human Genome

The nomenclature of the isoforms follows what was used in the NCBI RefSeq database and was described in “Materials and Methods”. For instance, the SIRT1 isoform-1 (termed SIRT1-v1, accession# NM_012238 for mRNA, and NP_036370 for protein) represented the sirtuin-1 (the homologs of the Sir2 protein), which had been widely studied for many years [13]. The other two SIRT1 isoforms were subsequently named SIRT1-v2 (accession# NM_001142498 for mRNA, and NP_001135970 for protein) and SIRT1-v3 (accession# NM_001314049 for mRNA, and NP_001300978 for protein), respectively. 

**Figure 1:**
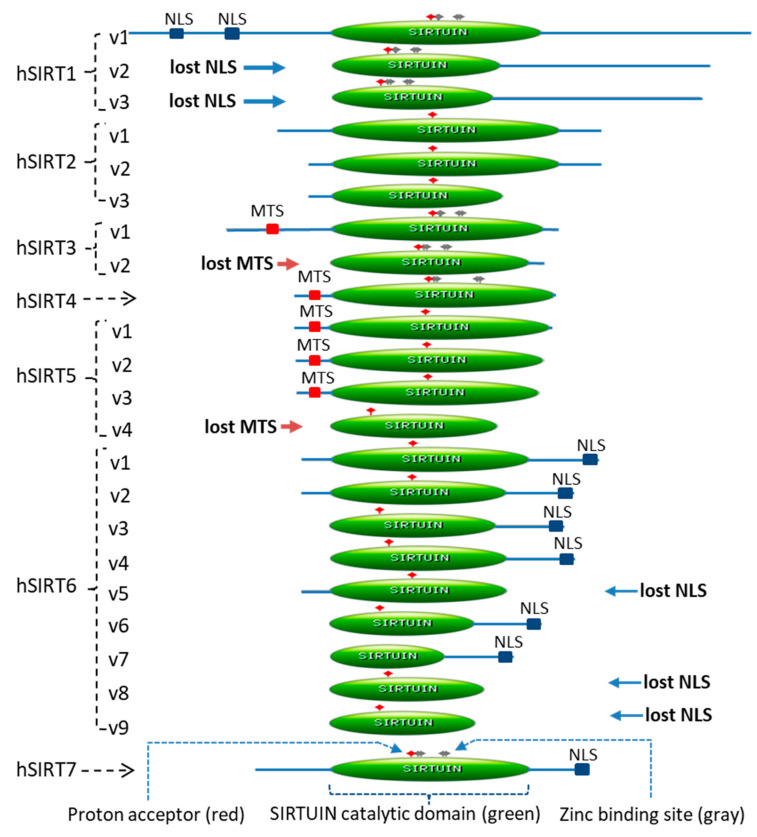
The twenty-three isoforms of human sirtuin genes (SIRT1-7). A schematic diagram of 23 isoforms. Most sirtuin isoforms lost part of the protein domains, which included part of the N- or C-terminus, catalytic domain (in green color), nuclear localization signal (NLS), and mitochondrial targeting signal (MTS). The functional domain analysis was performed using the “ScanProsite” software [16], which showed the catalytic domain “SIRTUIN” in green color, the “Proton acceptor” in red color, and the “Zinc binding site” in gray color. Here the SIRT7 protein was used as an example for detailed illustration, but the functional domain and sites are similar among all 23 isoforms.

There were 23 annotated human sirtuin isoforms in the RefSeq Database, which were derived from the seven sirtuin genes (SIRT1–SIRT7) that were located at different gene loci on different chromosomes (Table 1) [2,14]. Five of the seven sirtuin genes, excluding SIRT4 and SIRT7, had more than one isoform, whereas SIRT6 had nine isoforms (Table 1 and Figure 1). 

Exon skipping happened in the majority of the sirtuin isoforms (Table 1). However, both exon skipping and exon inclusion occurred in SIRT1-v2 and SIRT1-v3 isoforms. Overall, the isoform-1 of each sirtuin gene was longer than the alternatively spliced isoforms. The length of the isoform proteins ranged from 176 amino acids (e.g., SIRT6-v9) to 747 amino acids (e.g., SIRT1-v1) (Table 1). 

To determine the functional domain of all the sirtuin isoforms, the protein sequences of 23 isoforms were analyzed using the web-based software “ScanProsite” [15]. The sirtuin catalytic domain length ranged from 138 amino acids (e.g., SIRT6-v7) to 275 amino acids (e.g., SIRT2-v1). Exon skipping caused the isoforms to lose part of the catalytic domain, N-terminal, or the C-terminal domain (Figure 1). 

The 23 isoform protein sequences were also subjected to analysis for the presence of nuclear localization signal and/or the mitochondrial targeting sequence signal. The NLS was found in the SIRT1-v1, six of the nine SIRT6 isoforms and SIRT7 proteins (Figure 1). Alignment of the NLS sequences of SIRT1-v1, six SIRT6 isoforms and SIRT7 proteins revealed that they shared a common sequence “P·KX·KRK”. Due to exon skipping, SIRT1-v2 and v3 isoforms, SIRT6-v5, v8, and v9 isoforms lost the NLS (Figure 1). Examination of the mitochondrial targeting sequence revealed that the SIRT3-v2 isoform and the SIRT5-v4 isoform lost the MTS (Figure 1).

**Figure 2:**
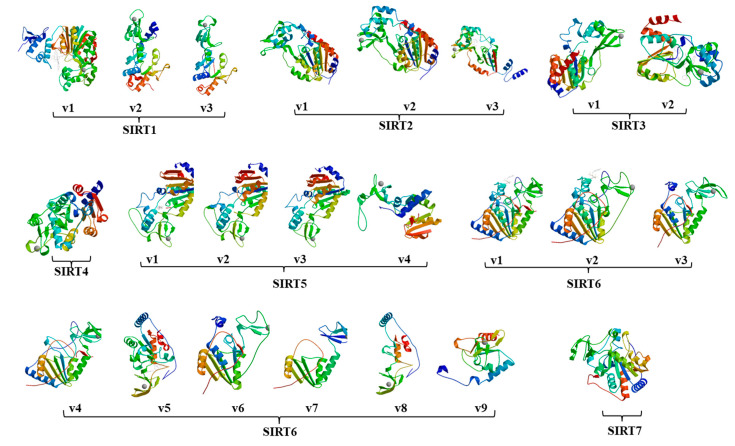
The 3-D sirtuin protein structure images that were generated using SWISS-MODEL software. Alternative splicing caused sirtuin protein domain loss and structural changes. Among the seven sirtuin genes, SIRT4 and SIRT5 genes had only one isoform each that was found in the RefSeq database.

To examine the potential structural changes due to the alternative splicing, all 23 sirtuin isoforms were subjected to protein structure homology-modeling using the SWISS-MODEL software [16]. As shown in Figure 2, protein domain loss resulted in minor to major structural changes among isoforms derived from the same sirtuin gene. For instance, loss of part of the N-terminus and part of the catalytic domain of the SIRT1-v2 and v3 isoforms changed their protein structures compared to SIRT1-v1 isoform. 

### 2.2. Sirtuin-1 (SIRT1) Gene Locus and Three Isoforms

**Figure 3:**
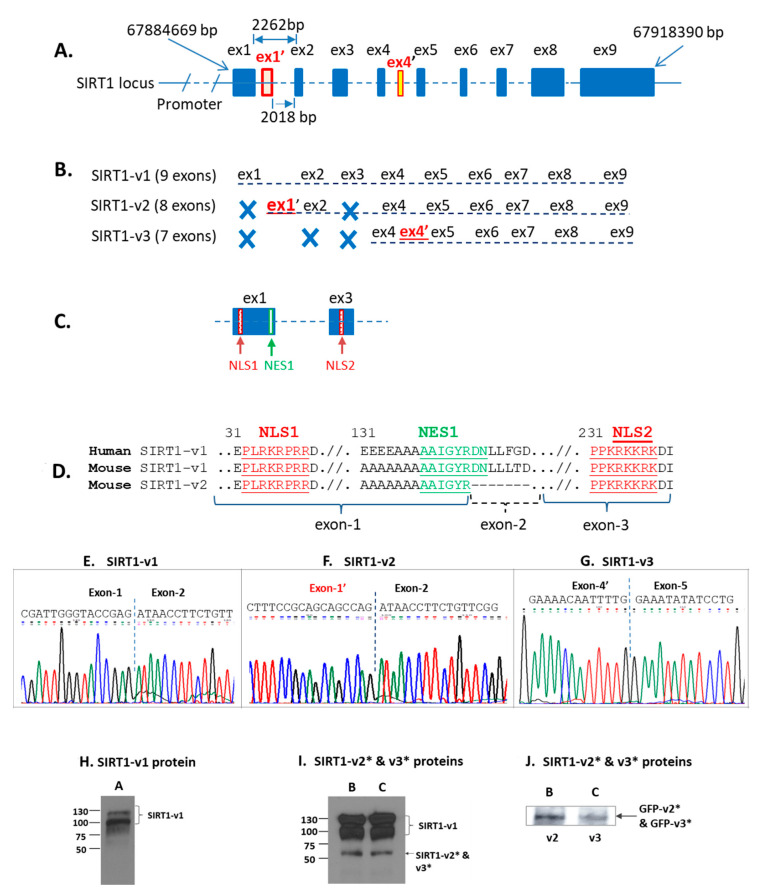
Bioinformatic and experimental analysis of human SIRT1-v1, v2, and v3 isoforms. (**A**). SIRT1 gene locus and genomic DNA landscape. (**B**). SIRT1-v1 isoform contained nine exons, whereas SIRT1-v2 contained eight exons, in which the first exon had not been previously reported and it was named exon-1′ (ex1′), SIRT1-v3 isoform contained seven exons. The Exon-4′ (ex4′) was immediately behind exon-4 and it had not been previously reported. (**C**). Exon-1 (ex1) and exon-3 (ex3) contained nuclear localization signal sequences. (**D**). Sequence analysis of human SIRT1-v1 in comparison with both mouse SIRT1-v1 and v2. During alternative splicing, mouse SIRT1-v2 lost exon-2, but retained exon-1 and exon-3 that have NLS. Therefore, both mouse SIRT1-v1 and v2 isoforms are likely to be localized in the nucleus. (**E**–**G**). The sequencing chromatogram image of exon-junctions that were observed in SIRT1-v1 (Figure 3E), SIRT1-v2 (Figure 3F), and SIRT1-v3 (Figure 3G). (**H**–**J**). SIRT1 isoform protein expression in cell lysate samples that were transfected with plasmid constructs SIRT1-v1 (**A**), SIRT1-v2 (**B**), and SIRT1-v3 (**C**). (**I**). SIRT1-v2 and v3 protein expression. (**J**). GFP-SIRT1-v2 and –v3 fusion proteins were detected by anti-GFP antibody. * The molecular weight of SIRT1-v2 is 50.5 kDa, and SIRT1-v3 is 49.25 kDa, respectively.

The human SIRT1 gene locus is located on chromosome 10 in the region ranging from 67884669 bp to 67918390 bp (accession# NC_000010). There are 11 exons in the genomic region, nine of which were previously reported and existed in the SIRT1-v1 isoform [17]. Two additional exons, termed exon-1′ (ex1′) and exon-4′ (ex4′) in this report, had not been previously reported (Figure 3A).

Both exon skipping and inclusion events happened to SIRT1-v2 and SIRT1-v3 isoforms. In SIRT1-v2 isoform, both exon-1 and exon-3 were skipped, but an extra exon (named exon-1′ or ex1′) was included during splicing, which was used as its first exon. Therefore, the SIRT1-v2 isoform (ex1′, ΔE1, ΔE2) contained a total of eight exons (Figure 3B). The SIRT1-v3 isoform (ΔE1, ΔE2, ΔE3, ex4′) contained seven exons, which skipped exon-1, 2, and 3, but included an extra exon (ex4′) (Figure 3B). Both exon-1 and exon-3 contains nuclear localization signal sequence, which is conserved between humans and mice (Figure 3C,D), skipping of these two exons could change the intracellular distribution of the isoform proteins. The ex1′ and ex4′ sequences were unique to isoform-2 and isoform-3, respectively (Figure 3A,B). Based on the sequence data, SIRT1-v1 isoform was the longest isoform with 747 amino acids in length, followed by SIRT1-v2 with 452 amino acids and SIRT1-v3 with 444 amino acids in length (Table 1). 

The catalytic domain of the human SIRT1-v1 is 254 amino acid in length (from 244 to 498 amino acid), which constituted 34% of the SIRT1-v1 protein. SIRT1-v2 and SIRT1-v3 isoforms, lacked part of the N-terminus compared to SIRT1-v1, and each had a shorter sirtuin domain (1/5 less) compared with that of SIRT1-v1 (Table 1 and Figure 1).

To confirm the presence of three SIRT1 isoforms in human tissues, the SIRT1 mRNA transcripts were amplified by RT-PCR, cloned and then sequenced. The chromatogram data revealed exon-exon junction of three SIRT1 isoforms. SIRT1-v1 had standard exon-1 and exon-2 junction (Figure 3E), SIRT1-v2 did not contain exon-1, instead, it had exon-1′ (Figure 3F), SIRT1-v3 lost exon-4 but had exon-4′ (Figure 3G). All three isoforms were found in the human heart and skeletal muscles. Our sequencing data were in agreement with those in RefSeq database. All three isoform proteins were also detected in SH-SY5Y cells transfected with SIRT1-v1, v2, and v3 in pReceiver-M29 expression plasmid vector, respectively, using immunoblotting (Figure 3H,I).

### 2.3. Differential Localization of Human SIRT1 Isoform Proteins in the Cells

Analysis of subcellular localization signals revealed that SIRT1-v1 had two NLS signal sequences and two nuclear export signal (NES) sequences. The NLS1 was located in exon-1 and the NLS2 was in exon-3 (Figure 3C), the NES1 was found in exon-1 and the NES2 was in exon-7, respectively. The SIRT1-v2 (ΔE1, ΔE3, ex1′) isoform lacked both exon-1 and exon-3, therefore, it did not have any NLS sequence. The SIRT1-v3 (ΔE1, ΔE2, ΔE3, ex4′) isoform lacked exon-1, 2, and 3, therefore, it did not have any NLS sequence either (Figure 3B,C). These data suggested that SIRT1-v2 and SIRT1-v3 proteins were likely to be present in the cytoplasm. 

Since the mouse SIRT1 gene has been well studied and mouse SIRT1 expression vectors have been widely used in the literature, it is worthwhile to compare the SIRT1 isoforms between humans and mice. The coding region of the mouse SIRT1 gene and its nuclear localization signal sequences were highly homologous with that of the human SIRT1 gene. There were two mouse SIRT1 isoform sequences in the RefSeq database (accession# NP_062786 for SIRT1-v1, and NP_001153061 for SIRT1-v2). The nuclear localization signal NLS1 was found in exon-1 and NLS2 in exon-3, NES1 in exon-1 and NES2 in exon-7 (Figure 3D). Both the mouse SIRT1-v1 isoform and SIRT1-v2 (ΔE2) isoform contained exon-1 and exon-3, which harbored NLS1 and NLS2. Therefore, both mouse SIRT1-v1 and v2 isoforms retained NLS1 and NLS2 sequences, which shared 100% homology to that of human SIRT1-v1 isoform (Figure 3D), suggesting that both mouse SIRT1 isoforms were likely to be localized in the nucleus. However, it is unclear whether the homologs of human SIRT1-v2 and v3 isoforms would exist in the mouse genome.

**Figure 4:**
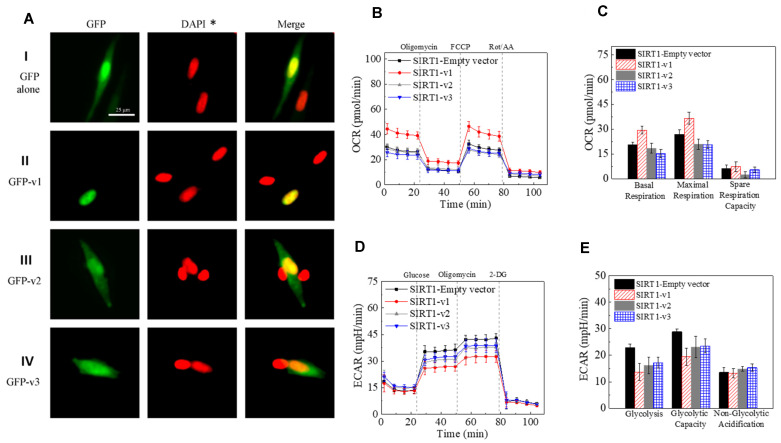
SIRT1 isoforms localization and functions. (**A**). Intracellular localization of GFP-SIRT1-v1, v2, and v3 fusion proteins in SH-SY5Y cells. GFP protein alone was shown in cytoplasm. GFP-v1 isoform protein was shown in nucleus. GFP-v2 protein was shown in cytoplasm. GFP-v3 isoform protein was shown in cytoplasm. The original DAPI was in blue color, the DAPI* here refers to the pseudo-color (red) that was converted from blue to red color using Nikon NIS-Elements Imaging Software. (**B**,**C**). SIRT1-v1 significantly increased the oxygen consumption rate (OCR) in term of basal respiration (*p* < 0.01, *n* = 3) and maximal respiration (*p* < 0.01, *n* = 3) compared to empty vector. Both SIRT1-v2 and v3 isoforms slightly reduced OCR compared with control (NS, *n* = 3). (**D**,**E**). The SIRT1-v1 significantly reduced glycolysis and glycolytic capacity compared with control (*p* < 0.01, *n* = 3). SIRT1-v2 and v3 also reduced glycolysis rate in the cells (*p* < 0.05, *n* = 3).

To visualize the intracellular localization of SIRT1 isoform proteins, three plasmids containing GFP and human SIRT1-v1, v2, and v3 isoform fusion protein were transfected into SH-SY5Y cells, respectively. As shown in Figure 4A, GFP alone was localized in cytoplasm, SIRT1-v1 was mainly localized within the nucleus, both SIRT1-v2 and SIRT1-v3 isoforms were mainly localized in the cytoplasm, which confirmed the bioinformatics analysis of protein localization.

### 2.4. The Effect of SIRT1 Isoforms Expression on Mitochondrial Function

To determine the effect of each SIRT1 isoform on mitochondrial function, the mitochondrial oxygen consumption rate and glycolysis rate were measured after overexpression of SIRT1-v1, v2, and v3 isoform plasmids in the cells, respectively. SIRT1-v1 significantly increased the oxygen consumption rate (OCR) in terms of basal respiration (*p* < 0.01, *n* = 3) and maximal respiration (*p* < 0.01, *n* = 3) compared to empty vector. Both SIRT1-v2 and v3 isoforms slightly reduced OCR compared with control (NS, *n* = 3) (Figure 4B,C). However, the SIRT1-v1 significantly reduced glycolysis and glycolytic capacity compared with control (*p* < 0.01, *n* = 3). SIRT1-v2 and v3 also reduced glycolysis rate in the cells (*p* < 0.05, *n* = 3) (Figure 4D,E). 

### 2.5. A Dynamic Expression Pattern of SIRT1 Isoforms in Human Tissues, HUVEC, and Mouse Hearts

**Figure 5:**
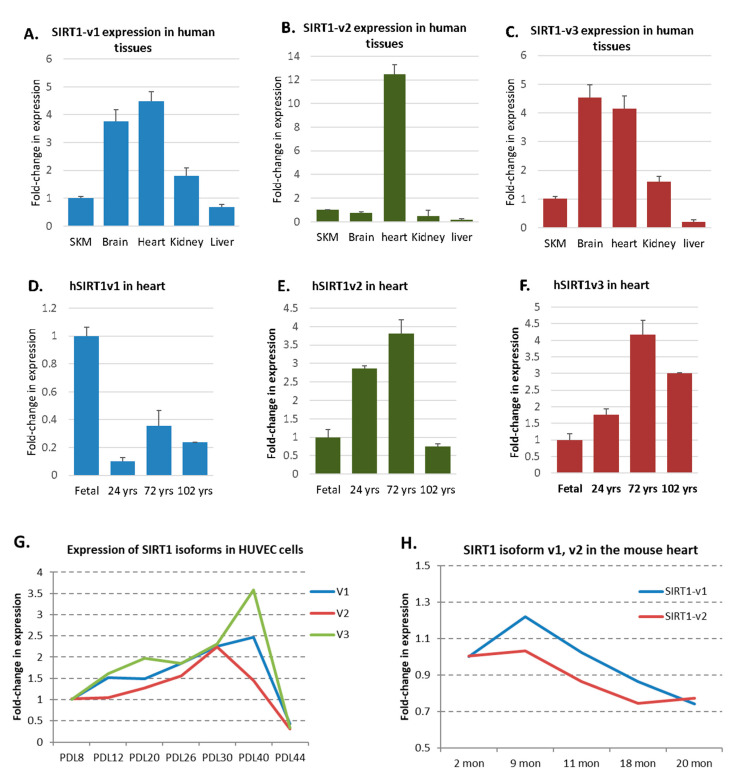
SIRT1 isoform expression in human and mouse hearts. (**A**–**C**). SIRT1-v1, v2, and v3 expression in human skeletal muscle (SKM), brain, heart, kidney and liver. (**D**–**F**). SIRT1-v1, v2 and v3 expression in human hearts of fetus, young adult, old and very old individuals. (**G**). SIRT1-v1, v2, and v3 expression in HUVEC cells at various multiple population doubling levels. (**H**). SIRT1-v1 and v2 expression in the mouse hearts from two months to 20 months of age.

To determine whether each of the three human SIRT1-v1, v2 and v3 may be differentially expressed in the human tissues, the isoform-specific PCR primers were employed. As shown in Figure 5A–C, the expression of the three SIRT1 isoforms in skeletal muscle (SKM), brain, heart, kidney and liver was measured, in which the SIRT1 isoforms were differentially expressed. The SIRT1 isoform expression was also measured in the human heart samples at various ages. SIRT1-v1 expression was at the highest level in the fetal heart, was decreased in the 24-year-old heart, slightly increased in the 72-year-old heart, and then decreased in 102-year-old heart (Figure 5D). The expression of isoforms SIRT1-v2 and SIRT1-v3 was at a low level in the fetal heart, but increased in 24 year old and 72 year old hearts, then decreased in 102 year old heart (Figure 5E,F). The expression of the SIRT1 isoforms was then measured in the HUVEC cells at different population doubling level (PDL). As shown in Figure 5G, all three isoforms were increased from PDL8 to PDL30, but gradually decreased around PDL44.

The expression of the SIRT1 gene was also measured in the mouse hearts at various ages. The mouse only had two SIRT1 isoforms. As shown in Figure 5H, SIRT1-v1 increased from the period of 2–9 months of age, but slightly declined at 20 months of age, whereas SIRT1-v2 declined from the period of 9–18 months of age, suggesting a dynamic expression pattern in the animals. 

## 3. Discussion

Aging is a complex biological process that is usually associated with a progressive decline in the biological functions of many tissues and organs in older adults. Multiple factors and various signaling pathways are likely involved in this dynamic life-long process. Alternative splicing tends to increase with advancing age, and alterations in alternative splicing may contribute to age-related changes and age-related disease [18,19,20].

Alternative splicing of pre-mRNA is an essential RNA processing mechanism that allows a single gene to generate two or more mature mRNA isoforms, thereby expanding the coding capacity of existing eukaryotic genomes, and increasing RNA and protein diversity. The alternatively spliced isoforms may include or exclude certain exons and translate into isoform proteins, which are slightly different from their constitutively spliced RNA and protein counterparts. These isoforms may have partially different RNA and protein sequences, and different intracellular localization or even distinct functions [21]. Alterations in alternative splicing have been observed in physiological and pathological conditions [22].

The sirtuins are a family of proteins that share a conserved domain of catalytic core with NAD^+^-dependent protein deacetylase activity. The catalytic core region contains a large domain that is homologous to the Rossmann-fold domain in sequence and structure, with a small Zn^2+^-binding domain and the cofactor binding loop region [23,24,25]. The Rossmann-fold domain has a conserved Gly-X-Gly sequence important for phosphate binding, a pocket to accommodate an NAD^+^ molecule, and charged amino acid residues responsible for ribose group binding, which shows the characteristics of NAD^+^-binding site [23,26,27]. The Zn^2+^-binding domain is a small but structurally diverse domain that consists of a three-stranded antiparallel β sheet and a variable α helical region, dependent on each sirtuin protein. The small domain contains the conserved sequence motif Cys-X2–4-Cys-X15–40-Cys-X2–4-Cys, a characteristic Zn^2+^-binding motif [23,27]. The cofactor binding loop region has four loops that link the small and large domains to form the cleft that acts as the enzyme active site. Both the NAD^+^ and acetyl-lysine substrates bind in this cleft [23,24]. It has been reported that the Rossmann-fold domain generally forms the bottom of the NAD^+^ binding site while the loop regions, mainly the cofactor binding loop, forms the rest of the pocket. When bound to a sirtuin enzyme, the NAD^+^ molecule adopts an extended conformation, which is commonly observed when NAD^+^ is bound to a protein having a Rossmann-fold [23,28]. Flanking the catalytic core are the N- and C-terminal regions, which host both NLS and MTS signal sequences and vary in length, sequence as well as secondary structure. These unique N- and C-terminal regions are important in mediating protein-protein interactions, intracellular localization, and likely contribute to altered biological processes mediated by sirtuin proteins. 

**Figure 6:**
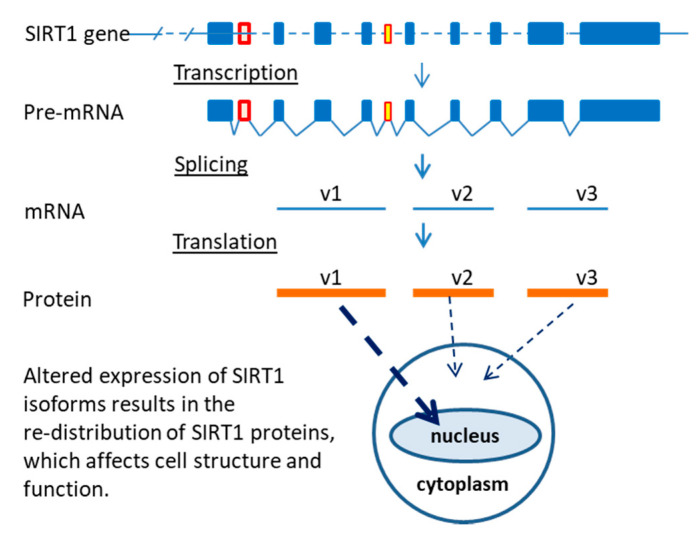
Schematic representation of the effect of alternative splicing on the localization of sirtuin isoform proteins. Human SIRT1-v1 isoform will be transported into the nucleus, whereas v2 and v3 isoform proteins will remain in cytoplasm. SIRT1-v1, v2, and v3 isoforms differentially regulate their target proteins and modulate cell structure and function, including mitochondrial function.

The alternative splicing in the sirtuin gene family that has been observed in the present study apparently results in exon skipping that generates isoforms with shorter sequences than the full-length protein. Some of them have lost NLS (e.g., SIRT1-v2 and v3, SIRT6-v5), while others have lost MTS (e.g., SIRT3). Within the SIRT1-v1 protein, the NLS sequence is located in N-terminal region, which is required for nuclear localization. The N-terminal region may also interact with SIRT1 regulators, including the active regulator of SIRT1 (AROS) protein [29]. At the C-terminal region, a 25 amino acid sequence is essential for SIRT1 activity (ESA) as a deacetylase [30,31]. These features suggest that splicing out of any exon either at N- or C-terminus, could affect SIRT1 function. The exon skipping in both SIRT1-v2 and v3 isoforms, generated two isoforms that were approximately 40% shorter than the full-length form, which not only produced two SIRT1 isoform proteins without an NLS signal but also shortened the N-terminal region that is crucial for proper protein-protein interactions. Therefore, the v2 and v3 isoforms were observed to be localized in the cytoplasmic and may interact with cytoplasm proteins and may be involved in other different signaling pathways (Figure 6). 

Exon skipping has also resulted in the loss of the crucial functional domain in other sirtuins. For instance, the isoforms that lost MTS would likely be excluded from the mitochondria. SIRT3, SIRT4, and SIRT5 are major regulators of mitochondrial oxidative phosphorylation [32,33]. The MTS sequence of SIRT3, SIRT4, and SIRT5 is located in the N-terminal region flanking the SIRT core domain (Figure 1). Exon skipping in the isoform SIRT3-v2 and SIRT5-v4 resulted in the loss of MTS, which are required for the SIRT3 and SIRT5 proteins to enter mitochondria. Likewise, NLS was found at the C-terminus of SIRT6 protein. The isoforms that lost NLS may be unable to enter the nucleus, which include SIRT6-v5, SIRT6-v8, and SIRT6-v9. 

Alternative splicing occurs in approximately 95% of all human genes and 60% of all mouse genes. The human and mouse genomes share similar long-range sequence organization, and most of the coding sequences are highly conserved and homologous. The intron sequences tend to have lower homology among species [34,35]. Similarly, although constitutive splicing events have a high level of conservation, the alternative splicing junctions usually bear a lower level of conservation across species [33,34]. A sizable fraction of alternative splicing events appears to be species-specific among different species, including humans, rodents, or other mammals. The alternative splicing events could also be lineage-specific and tissue-specific [36]. It is estimated that approximately one-third of the alternative splicing junctions observed in humans are apparently not conserved in the mouse genome [34,35,36]. 

Both human and mouse genomes contain seven sirtuin genes. To date, 23 human sirtuin isoforms and 15 mouse sirtuin isoforms have been annotated in the RefSeq database (Data not shown). In addition, several alternatively spliced sirtuin isoforms have been reported. For instance, a SIRT1-ΔExon8 has lost exon-8 therefore only has weak deacetylase activity [37]. In addition, a novel splice variant (isoform-5) of SIRT2, which lacks a nuclear export signal, is essentially localized within the nucleus [38]. In the present study, human SIRT1-v2 and SIRT1-v3 isoforms have apparently lost the NLS, while the mouse SIRT1-v1 and SIRT1-v2 isoforms retained NLS sequence. This tends to support the notion that the sirtuin isoforms observed in the human are not completely conserved in the mouse [36]. As a consequence, alternative splicing adds to the complexity when applying sirtuin inhibitors and activators to different models across species [39]. Furthermore, the data from mouse models with sirtuin gene manipulations may not be readily translatable to clinical applications. 

Taken together, we have identified 23 human sirtuin isoforms and 15 mouse sirtuin isoforms in this report. Each alternatively spliced sirtuin isoform represents a variant protein that differs from the constitutively spliced protein in sequences, which would increase the sirtuin gene diversity and modulate their subcellular localization and function. Although each isoform may have a different and possibly minor effect on cellular function, the combined effects of the altered expression of all 23 sirtuin isoforms could have a profound effect on an array of physiological functions of multiple tissues and organs. The tissue-specific and developmental stage-specific expression of the sirtuin isoforms warrant further investigation. In addition, the splicing events of sirtuin genes were not completely conserved between mice and humans, which would further add complexity to attempts to extrapolate animal data to human physiology. A comprehensive study of alternative splicing and various isoforms during development, maturation, and advancing age would help to enhance our understanding towards adult aging and senescence.

## 4. Materials and Methods

### 4.1. The Nomenclature of the Sirtuin Isoforms and Bioinformatics Analysis

The nomenclature of the sirtuin isoforms follows what is used in The National Center for Biotechnology Information (NCBI) Reference Sequence (RefSeq) database, in which the constitutively spliced form of sirtuin gene is termed as isoform-1, and alternatively spliced isoforms are named as isoform-2, isoform-3, etc. [14]. The data collection was completed as of 30 June 2020. 

All the sirtuin gene genomic sequences and mRNA isoform sequences were obtained from RefSeq database [14]. The gene sequence analyses were performed using the following software programs: The web-based software “Blast” and “MultAlin” were used for sequence alignment [40], the “ScanProsite” software was used to determine the conserved domain of the sirtuin isoform [15], the SWISS-MODEL software was used for analysis of the 3-D protein structure [16]. The software NLS Mapper [41], NLSdb [42], NetNES [43] and LocNES [44] were used for the analysis of nuclear localization signal (NLS) and the nuclear exporting signal (NES) sequences. The software MITOPROTII [45], TargetP [46] and MitoFates [47] were employed for the analysis of mitochondrial targeting sequence (MTS).

### 4.2. Human and Mouse Tissues, mRNA and cDNA Samples

The studies were conducted with Institutional Review Board approval from the University of Arkansas for Medical Sciences, in accordance with the NIH Guiding Principles for Research Involving Animals and Human Beings. The animal use protocol was approved by the Central Arkansas Veterans Healthcare System IACUC, Little Rock, AR on 24 June 2019 under IACUC #1238531.

The majority of the human fetal and adult heart mRNA, as well as cDNA samples, was obtained from BioChain Institute (Hayward, CA, USA), Clontech (Mountain View, CA, USA), and Zyagen (San Diego, CA, USA), including the human fetal (20–27 weeks of age) heart samples, human adult (21, 51, 32, and 91 years of old) heart, brain, kidney, liver, colon, and skeletal muscle samples. We also utilized some heart mRNA and cDNA samples that were used in our previous study [20]. C57BL/6 mouse hearts were obtained from Rodent and Tissue Bank from the National Institute of Aging (NIA). 

### 4.3. Cell Culture, Plasmid Constructs and Quantitative Reverse-Transcriptase PCR

The SH-SY5Y cell line (ATCC CRL-2266) was purchased from ATCC (Manassas, VA, USA). Human umbilical vein endothelial cells (HUVECs) were obtained from Lonza (Walkersville, MD, USA), as previously described [48]. The cell growth reagents and media (DMEM, DMEM/F12), newborn bovine serum, lipofetamin 2000 were purchased from Invitrogen (Carlsbad, CA, USA), ATCC, and Lonza, respectively.

The cDNA samples of the human heart, skeletal muscle, and HT-29 cell line were used for the amplification of SIRT1 isoforms. The amplified isoforms were subcloned into pGEM-T Easy Vector Systems and then sequenced using T7 and sp6 primers. The GFP and human SIRT1-v1, v2, and v3 isoform fusion cDNA sequences were in pReceiver-M29 expression plasmid vector under the control of CMV promoter (Genecopoeia, Rockville, MD, USA). The sequences of all the plasmid constructs were verified by sequencing analysis. 

The isoform-specific primers were selected from the unique sequence region of each isoform sequence using Primer-Blast (NCBI) and synthesized by Integrated DNA Technologies (Coralville, IA, USA). The PCR primers for the detection of human genes are as follows: SIRT1-v1 forward primer: gagggcgaggaggaggaagag, reverse primer: gtccagtcactagagcttgca. SIRT1-v2 isoform forward primer: ttcgctcttttcctccgtcc, and reverse primer: acagaaggttatctggctgct. SIRT1-v3 isoform forward primer: ctgtgcagtggaaggaaaaca, and reverse primer: gattcccgcaacctgttcca. Mouse SIRT1-v1 isoform forward primer: ttgaccgatggactcctcac, reverse primer: gtcactagagctggcgtgtg. Mouse SIRT1-v2 isoform forward primer: cggctaccgaggtccatatac, reverse primer: agctcaggtggaggaattgt. The quantitative RT-PCR was performed as previously described [49].

### 4.4. Measurement of Mitochondrial Oxygen Consumption and Glycolytic Rate

SH-SY5Y cells were seeded at approximately 60,000 cells per well in XF96 Well plates (Seahorse Bioscience, Billerica, MA, USA). The cells were transfected with eGFP-empty vector plasmid or the plasmid containing eGFP-SIRT1-v1, v2, or v3 fusion gene for 24 h, respectively. Then the cells were subjected to extracellular flux analysis using the XF Cell Mito Stress Test Kit and XF Glycolytic Rate Assay Kit, respectively (Seahorse Biosciences, Billerica, MA, USA). The measurement was performed as previously described [48].

### 4.5. Immunocytochemical Analysis

Briefly, the expression plasmid constructs containing empty vector and SIRT1 isoforms were transfected into SH-SY5Y cells using Lipofectamine 2000 (Invitrogen). At 24 h after the transfection, the cells were subjected to imaging analysis using a Nikon C2si confocal microscope (Nikon, Inc., Melville, NY, USA) ), as previously described [50].

### 4.6. Western Blotting

Western blotting was conducted as previously described [48]. In brief, the SH-SY5Y cells were transfected with SIRT1-v1, v2, and v3 in pReceiver-M29 expression plasmid vector, respectively. At 24 h after transfection, the cells were lysed in a lysis buffer (10 mM Tris, pH 7.4, 100 mM NaCl, 1 mM EDTA, 1 mM ethylene glycol tetraacetic acid, 1% Triton X-100, 10% glycerol, and 0.1% sodium dodecyl sulfate) supplemented with 1× protease inhibitor cocktail (Sigma-Aldrich, St Louis, MO, USA). Proteins were denatured in Laemmli sample buffer for 5 min at 95 °C and resolved by Mini-PROTEAN TGX gels (Bio-Rad, Hercules, CA, USA). The primary antibodies were purchased from Cell Signaling Technology (anti-SIRT1), Thermo Fisher Scientific (anti-GFP). The secondary antibodies were purchased from Bio-Rad Laboratories. 

### 4.7. Statistical Analysis

Data are given as mean values ± SD, with n denoting the number of experiments unless otherwise indicated. A two-tailed *T*-test was used to determine the differences between the two groups. A *p*-value of <0.05 was considered to be statistically significant.

## Figures and Tables

**Table 1:** A summary of 23 human sirtuin isoform mRNAs and proteins and their Genbank accession number. Alternative splicing resulted in the loss of part of the sequence and shortened the length of the sirtuin domain of most of the isoforms.

Gene	Chromosome	Isoform	mRNA Accession#	Exon-Skipping	Protein Accession#	Amino Acid	Sirtuin Domain		
							from–to	Length	% (SIRT/Total)
SIRT1	10	V1	NM_012238		NP_036370	747	244–498	254	34.0
		V2	NM_001142498	ex1, ex3	NP_001135970	452	1–203	202	44.7
		V3	NM_001314049	ex1, ex2, ex3	NP_001300978	444	1–195	194	43.7
SIRT2	19	V1	NM_012237		NP_036369	389	65–340	275	70.7
		V2	NM_030593	ex2	NP_085096	352	28–303	275	78.1
		V3	NM_001193286	ex2, ex13, ex14	NP_001180215	234	28–234	206	88.0
SIRT3	11	V1	NM_012239		NP_036371	399	126–382	256	64.2
		V2	NM_001017524	ex2	NP_001017524	257	1–240	239	93.0
SIRT4	12	V1	NM_012240		NP_036372	314	45–314	269	85.7
SIRT5	6	V1	NM_012241		NP_036373	310	41–309	268	86.5
		V2	NM_031244	ex1, ex10	NP_112534	299	41–299	258	86.3
		V3	NM_001193267	ex1, ex7	NP_001180196	292	41–291	250	85.6
		V4	NM_001242827	ex1, ex4	NP_001229756	202	1–202	201	99.5
SIRT6	19	V1	NM_016539		NP_057623	355	35–274	239	67.3
		V2	NM_001193285	ex6	NP_001180214	328	35–247	212	64.6
		V3	NM_001321058	ex2	NP_001307987	283	1–202	201	71.0
		V4	NM_001321059	ex3	NP_001307988	294	1–213	212	72.1
		V5	NM_001321060	ex7	NP_001307989	248	35–248	213	85.9
		V6	NM_001321061	ex2, ex6	NP_001307990	256	1–175	174	68.0
		V7	NM_001321062	ex2, 3x3	NP_001307991	220	1–139	138	62.7
		V8	NM_001321063	ex3, ex7	NP_001307992	187	1–187	186	99.5
		V9	NM_001321064	ex1, ex7	NP_001307993	176	1–176	175	99.4
SIRT7	17	V1	NM_016538		NP_057622	400	90–331	241	60.3
